# A Patterned Butyl Methacrylate-*co*-2-Hydroxyethyl Acrylate Copolymer with Softening Surface and Swelling Capacity

**DOI:** 10.3390/polym11020290

**Published:** 2019-02-09

**Authors:** Laia León-Boigues, Catalina von Bilderling, Lía I. Pietrasanta, Omar Azzaroni, Juan M. Giussi, Carmen Mijangos

**Affiliations:** 1 Instituto de Ciencia y Tecnología de Polímeros, ICTP-CSIC, Juan de la Cierva 3, 28006 Madrid, Spain; laleboi@ictp.csic.es; 2 Instituto de Investigaciones Fisicoquímicas Teóricas y Aplicadas (INIFTA)–Departamento de Química–Facultad de Ciencias Exactas-Universidad Nacional de La Plata–CONICET, 1900 La Plata, Argentina; catalinavb@inifta.unlp.edu.ar (C.v.B.); azzaroni@inifta.unlp.edu.ar (O.A.); 3 Departamento de Física, Facultad de Ciencias Exactas y Naturales, Universidad de Buenos Aires, C1428EHA Buenos Aires, Argentina; lia@df.uba.ar; 4 Instituto de Física de Buenos Aires (IFIBA-CONICET), C1428EHA Buenos Aires, Argentina; 5 Donostia International Physics Center (DIPC), Paseo Manuel Lardizabal 4 and Centro de Fisica de Materiales, CFM-CSIC/UPV-EHU Paseo de Manuel Lardizabal 5, 20018 Donostia-San Sebastian, Spain

**Keywords:** anodic aluminum oxide template, free radical copolymerization, swelling and mechanical properties

## Abstract

The tunable swelling and mechanical properties of nanostructures polymers are crucial parameters for the creation of adaptive devices to be used in diverse fields, such as drug delivery, nanomedicine, and tissue engineering. We present the use of anodic aluminum oxide templates as a nanoreactor to copolymerize butyl methacrylate and 2-hydroxyethyl acrylate under radical conditions. The copolymer obtained under confinement showed significant differences with respect to the same copolymer obtained in bulk conditions. Molecular weights, molecular weight dispersities, Young’s modulus, and wetting behaviors were significantly modified. The combination of selected monomers allowed us to obtain nanopillar structures with an interesting softening surface and extraordinary swelling capacity that could be of special interest to surface science and specifically, cell culture.

## 1. Introduction

The rapid development of polymer science for nanotechnology requires the combination of well-defined sub-micron scale structures and specific chemical functionalities. Cylindrical nanocavities of anodic aluminum oxide (AAO) are an ideal matrix system to prepare well-defined sub-micron scale polymeric structures, i.e., polymer nanostructures. A simple copolymerization process in which different chemical structures are present in a single copolymer chain provides an illustrative example of polymers with specific and dual chemical functionalities. 

Aligned cylindrical nanocavities of self-ordered AAO templates with rigid walls have been applied for the easy and high-throughput nanomolding of a wide range of polymer nanoarchitectures [1], nanopillars [2], nanofibers [3], nanotubes [4], and nanospheres [5] over large areas. In addition, these nanostructures feature novel characteristics with respect to their non-nanomolding analogous, and these new properties generally improve the material potentiality. The traditional and most widely used method to prepare polymer nanoarchitectures using AAO templates is the melting procedure. According to this procedure, a powdered or film polymer is infiltrated into the nanocavities at high temperature and/or for a long time [6]. Nonetheless, the use of nanomolding in melting procedures is limited by polymer degradation and by being time-consuming.

An alternative method to design polymer nanostructures is the in-situ polymerization of a polymer precursor monomer into AAO nanocavities. So far, the advantage of polymer nanofabrication within AAO nanocavities has been demonstrated as a straightforward pattern of polymer nanostructures in the case of the in-situ free radical polymerization of styrene [7], methyl methacrylate [8], or fluoracrylic monomers [9]. In all cases, high reaction conversion with controllable molecular weight and molecular weight dispersities, in comparison to bulk polymerization in the same conditions, was reached in a few hours. In addition, the step-growth polymerization process was successfully carried out, until reaching almost 100% of conversion in less than 3 h. Moreover, the atom transfer radical polymerization technique [10] was demonstrated to be a feasible process in AAO templates. In short, the in-situ synthesis of polymers is a generalized process of polymerization reactions at a nanoscale, being, in all cases under study, a faster and less energetic process than the polymer infiltration process itself. An interesting outcome is that, in some cases, the effect of polymer nanostructuration was found to promote cell proliferation [11,12].

In addition, it should be borne in mind that certain processes can only occur if the correct mechanical features are selected [13]. Moreover, the nanomechanical properties of materials are a crucial factor in the design of adaptive devices [14,15], and the surface properties of soft platforms are key players in the development of biomedical materials [16]. In this regard, amphiphilic copolymers obtained using hydrophilic and hydrophobic monomers have demonstrated good application in areas such as biomaterials [17], coatings [18], petroleum science [19], and more. In this context, hydroxyethyl acrylate (HEA) is a soft hydrophilic monomer extensively used in biomedical applications, such as controlled release [20], and hemotherapy [21] to name a few.

Free radical copolymerization in confinement has never been used to obtain nanostructures with tunable properties. Due to this fact, the intention of this exploratory work was two-fold: on the one hand, to report to study the free radical copolymerization of butyl methacrylate (BMA) and 2-hydroxyethyl acrylate (HEA) in confinement using AAO nanoreactors, and, on the other, to obtain nanostructures with tunable mechanical characteristics and swelling/wetting properties. To meet these goals, we combined free radical copolymerization with AAO template synthesis; and used nuclear magnetic resonance to identify the copolymer composition. Size exclusion chromatography was performed to obtain the molecular weight of copolymers and scanning electron microscopy to evidence the nanostructures obtained. Additionally, atomic force microscopy and water contact angle measurements were performed to determine mechanical properties and wettability characteristics of copolymer nanostructures. The contribution in this paper can be seen as a step forward in copolymer synthesis in confinement with high polymer conversion and polydispersity index (PDI) similar to that of a controlled polymerization (living), being a free radical polymerization. Additionally, the copolymer showed a significant improvement regarding its swelling and wetting properties and interesting softening in comparison with bulk polymers. The mechanical properties, as well as the swelling and wetting characteristics of these nanopillar structures, could be applicable to several processes, including scaffolds for cell culture.

## 2. Methods and Materials

### 2.1. Copolymer Synthesis

*Confined copolymerization.* AAO template was hard washing with different solvents in ultrasound and dry in an oven at high temperature. 6 ml solution of butyl methacrylate and 2-hydroxyethyl acrylate monomers was prepared using an initial monomer molar composition of 0.45 for BMA and 0.55 for HEA (fBMA = 0.45 and fHEA = 0.55) with AIBN (0.47% w/v) as initiator. The solution was introduced in a round bottom flask and purged during 15 minutes under nitrogen bubbling. Then, AAO template was introduced into the round bottom flask under vacuum for 30 minutes. Afterwards, AAO template was retired from the flask and placed into an oven at 40 °C increasing the temperature 10 °C every 20 minutes to 70 °C. The sample was allowed to react into the oven during 24 h at 70 °C. After that, the AAO template was retired and superficially cleaned to remove unconfined copolymer layer on the template surface. Monomer conversion = 100% (from spectrometry)

*Bulk copolymerization.* 6 mL solution of butyl methacrylate and 2-hydroxyethyl acrylate monomers was prepared using an initial monomer molar composition of 0.45 for BMA and 0.55 for HEA (fBMA = 0.45 and fHEA = 0.55) with AIBN (0.47% w/v) as initiator. The solution was introduced in a round bottom flask and purged during 15 minutes under nitrogen bubbling. The copolymerization was carried out at 70 °C during 24 h, the same conditions respect to confined copolymerization and then it was introduced into ice to stop the reaction. The copolymer was purified by three steps of dissolutions in chloroform and precipitation in methanol, centrifuged and dried under vacuum. Monomer conversion = 72% (from gravimetry).

### 2.2. Copolymer Characterization

*SEM Characterization.* The AAO templates and nanopillars obtained were morphologically characterized by scanning electron microscopy (SEM) (Hitachi, 8100, Hitachi High-Technologies Europe GmbH, Krefeld, Germany). In order to perform the analysis of free nanopillars, the aluminum substrate was treated with a mixture of HCl, CuCl_2_, and H_2_O and the alumina was dissolved in 10% wt H_3_PO_4_. Previously, in order to support the free nanostructures, a coating was placed over the template. 

*NMR and GPC Characterization.* The copolymer was characterized by nuclear magnetic resonance (Bruker 300 MHz, Santa Barbara, USA) using deuterated. Gel permeation chromatography (GPC) analyses were carried out with Styragel (300*7.8 mm, 5mm nominal particle size) Water columns. THF was used as a solvent. Measurements were performed at 35 °C at a flow rate of 1 mL/min using an RI detector. Molecular weights of polymers were referenced to PS standards. MW range covered was from 5,000 to 2,000,000. In order to perform the analysis, the samples were treated as is explained below. 

Confined copolymer: After the reaction, the copolymer was extracted from AAO templates by submerging the template in a vial with chloroform or THF (depending on employed characterization technique, NMR or GPC, respectively) and stirred during two days. Afterwards, placed in an ultrasound bath for several periods of 2 h. Then, for NMR characterization, the solution goes directly to be analysed, and for GPC characterization, the solution was filtered, precipitated in methanol and dissolved again in THF before going to GPC.

Bulk copolymer: After the reaction, the bulk copolymer obtained was dissolved in chloroform or THF (depending on characterization technique, like in confined process) and stirred during few hours. Then, the solution goes to the equipment directly to be analysed.

*IR Characterization*. The Fourier transform infrared (FTIR) spectra of the copolymer obtained in bulk conditions was measured using KBr pellet method. ATR-FTIR method was performed for confined copolymer. In this case, the measurement was done with the copolymer inside the template. The measurements were made between 4000–800 cm^−1^ with a resolution of 4 cm^−1^ and 32 accumulated scans. 

*Atomic Force Microscopy.* AFM measurements were performed in dry nitrogen or aqueous (milliQ) environment using a Multimode 8 AFM (Nanoscope V Controller, Bruker, Santa Barbara, CA, USA). Peak force tapping was selected as the imaging mode. V-shaped AFM probes from Bruker were used: Scanasyst-air (0.4 N/m cantilever nominal spring constant) for dry measurements and SNML (0.07 N/m cantilever nominal spring constant) for liquid measurements. For elasticity measurements, the spring constants of the cantilevers were determined for each experiment by the thermal tune method [22], and the deflection sensitivity was determined in fluid using freshly cleaved mica as a stiff reference material. Force curves were acquired using force volume mode, for which a force curve is performed at each pixel in a map. From force volume maps of 32 × 32 pixels on a 2 µm^2^ area, all force curves were analyzed to quantify the Young moduli distributions. Tip shape was estimated using the blind estimation method using a titanium roughness sample (Bruker, Santa Barbara, CA, USA). The observed radius of curvature of the tips was ~20 nm. Stiffness was obtained using the Oliver and Pharr method [23,24], through the slope of each curve calculated by performing a linear fit to the upper part of the retraction force curve. The Poisson’s ratio was assumed to be 0.5. Image processing was performed using the commercial Nanoscope Analysis software (Bruker, Santa Barbara, CA, USA). Young modulus was obtained from force curves through custom written Matlab (Mathworks, Natick, MA, USA) routines by using the Bruker Matlab Utilities package. To perform the analysis of free nanopillars, the same procedure described for SEM studies was performed.

*Water Contact Angle*. Contact angle measurements were carried out using a KSV theta goniometer (Succasunna, NJ, USA) with deionized water. In a typical measurement, 7 μL droplet of water was deposited on the sample surface. The average contact value was obtained at five different positions of the same sample. To perform the analysis of free nanopillars, the same procedure described for SEM studies was performed. 

## 3. Results and Discussion

**Figure 1:**
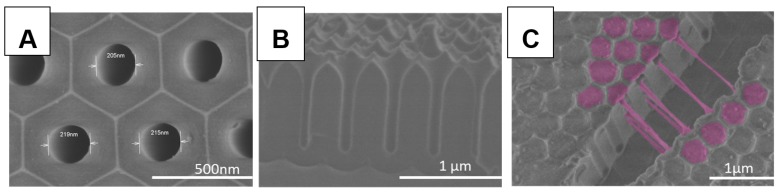
SEM images from (**A**) Top view and (**B**) Lateral view of synthesized anodic aluminium oxide (AAO) template. (**C**) Top view of a cracked template after in-situ polymerization of 2-hydroxyethyl acrylate (HEA) and butyl methacrylate (BMA).

**Figure 2:**
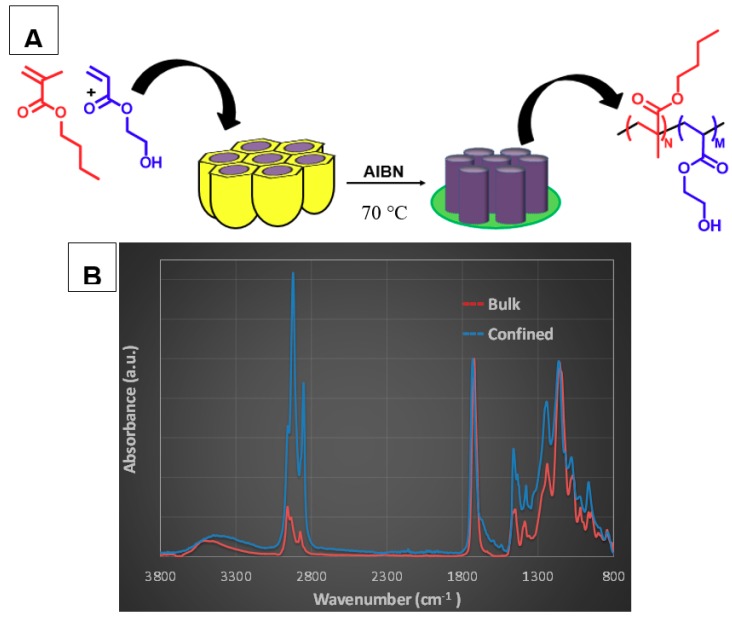
(**A**) Schematic copolymerization procedure of BMA and HEA monomers under confinement, and monomers and copolymer structures. (**B**) Infrared spectroscopy (IR) spectra of the copolymers obtained under confinement and in bulk conditions.

Firstly, based on Masuda et al. [25], AAO templates were prepared following a two-step anodization process to achieve well-ordered pore structures. Pore size and length were controlled by adjusting the synthesis parameters to obtain a well-defined geometry. As shown in Figure 1, we prepared AAO templates with 200 nm pore diameter (Figure 1A) and 1µm pore length (Figure 1B). Afterwards, butyl methacrylate (BMA) and 2-hydroxyethyl acrylate (HEA) were copolymerized inside these pores using 2,2’-Azobis-(isobutyronitrile) as initiator, as illustrated in Figure 2A (see supporting information for experimental details).

Figure 1C corresponds to SEM images of a cracked AAO template after polymerization (the Figure shows the template top view). As it can be observed, the surface of the nanocavities is a polymer-free zone. In the crack region, the filled pores with the polymer in all the nanocavities can be clearly seen. In addition, a good polymer distribution along the pores is noticed from the bottom to the surface. Regarding Figure 1C, the copolymer obtained exhibits excellent elasticity and flexibility (see Figure S1 in Supplementary Materials for further details).

The copolymers were extensively characterized by nuclear magnetic resonance (NMR) and infrared spectroscopy (IR). The IR spectra of the copolymers synthesized in bulk and in confinement displayed the typical signal 2930 and 2850 cm^−1^ (C–H, Aliphatic), 1700–1730 cm^−1^(C=O, ester), 1050 and 1290 cm^−1^ (C–O, ester). The absence of C=C stretching peak at 1550 cm^−1^ in Figure 2B indicates a complete reaction for confinement copolymerization with a conversion close to 100% with respect to 72% for bulk condition (estimated by gravimetry). The extender NMR spectra of homo and copolymers obtained in bulk with assignments of resonance peaks are illustrated in Figure S2. The H NMR showed all signals corresponding to copolymer signals, and no monomer or impurities signals. The final copolymer composition was estimated through the H NMR spectra, as indicated in the Supplementary Materials, and the values obtained were 0.47 for BMA and 0.53 for HEA (F_BMA_ = 0.47 ± 0.3 and F_HEA_ = 0.53 ± 0.2) in copolymerization under confinement and 0.62 for BMA and 0.38 for HEA (F_BMA_ = 0.62 ± 0.5 and F_HEA_ = 0.38 ± 0.3) in copolymers obtained under bulk conditions. As is known, the reactivity ratios for the BMA/HEA system [26], indicate that BMA should be preferentially incorporated into the copolymer at the start of the batch reaction. However, the extent of BMA enrichment will decrease with overall conversion: at 100% monomer conversion in the confined synthesis, the average copolymer composition is 0.47 for BMA and 0.53 for HEA, matching that of the original comonomer mixture. 

The copolymers obtained displayed interesting differences. The Mn value of the copolymer obtained in confinement was 18,180 g/mol eq PS (PDI = 1.6), considerably lower than the one obtained in bulk, 442,430 g/mol eq PS (PDI = 2.2). According to Sanz et al., the molecular weight decreases in confinement due to an increase in the k_d_ value [8]. These authors confirmed a faster decomposition of azobisisobutyronitrile (AIBN) within the AAO templates due to a catalytic effect of the pore walls on the initiator decomposition. This effect produces higher decomposition efficiency and results in a higher number of radical species and, therefore, greater chain growth that leads to a decrease in the molecular weight of the polymer obtained in the AAO templates. Additionally, according to theoretical predictions [27], as the PDI value is less than two, the results also suggest that, in confined copolymerization, the combination would be the predominant termination mode, while, in bulk copolymerization, as the PDI value close to two, chain transfer or termination by disproportionation would occur.

**Figure 3:**
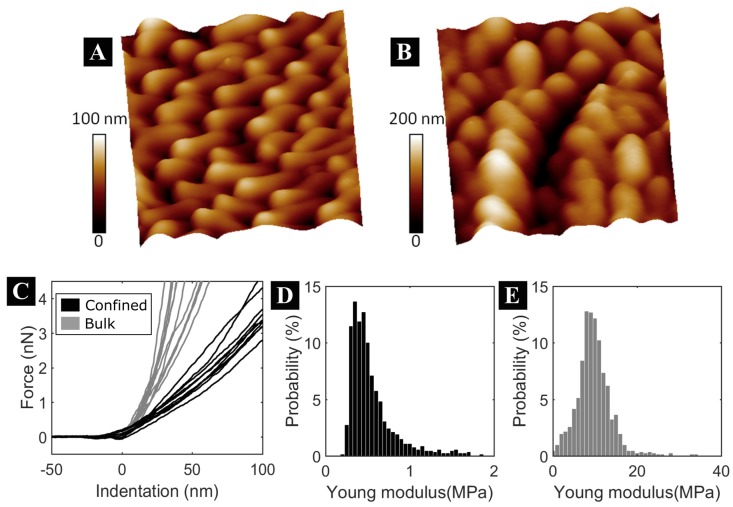
(**A**–**B**) In-situ atomic force microscopy (AFM) three-dimensional topography images (2.5 µm × 2.5 µm) of free nanopillars of the copolymer synthesized in confinement in a dry environment (**A**) and an aqueous environment (**B**). (**C**) Typical AFM force vs. indentation curves for free copolymer nanopillars (black) and bulk films (gray). (**D**–**E**) Young’s Modulus (*E*) histograms from N > 900 force curves of copolymers synthesized in confinement (**D**, *E* = 0.54 ± 0.26 MPa) and in bulk (**E**, *E* = 9.9 ± 5.8 MPa), standard deviations taken as error. Horizontal scales are different as they were optimized for each distribution.

Swelling, wettability and mechanical properties of the copolymers obtained were investigated for their potential applications. As known, the swelling and wettability capacities are primary characteristics when it comes to cell culture. Additionally, cells respond to substrate stiffness, since this parameter may influence cell adhesion and proliferation. To determine the swelling and mechanical properties of the nanopillars obtained, atomic force microscopy (AFM) measurements were performed on free nanopillars of the copolymer synthesized in confinement and on the films of the copolymer synthesized in bulk. The treatment to obtain the free nanopillars and bulk films is explained in Supplementary Material. The swelling and mechanical properties of the copolymers obtained displayed interesting and promising differences. Figure 3 illustrates the AFM topography images (2.5 µm × 2.5 µm) of the copolymer synthesized in confinement. Figure 3A corresponds to the measurement in a dry environment and in an aqueous environment (previously, the sample was kept for half an hour in water to reach swelling equilibrium). Water influence on the nanopillar sizes can be observed. The swelling effect of these nanomaterials showed an important size variation. The nanopillar diameters were estimated as the average value of 8 nanopillars. The swelling produces a diameter size that ranges from (180 ± 30) to (270 ± 65) nm. The percentage of volume increase, estimated as $100\times \left(r_{2}^{2}/r_{1}^{2}\right)$, is of 225%.

The nanomechanical properties of the bulk copolymer film and the nanopillars in a swollen state were examined with AFM, a technique that has been successfully applied to several processes for this same purpose [28,29,30]. Figure 3C shows typical AFM force vs indentation curves obtained for the nanomechanical analysis, from which elasticity differences between the samples are evident: For a loading force of 4 nN, the nanopillar indentation is of about 100 nm, while in bulk copolymer film indentation is less than 50 nm at the same force amplitude. The frequency histograms of the Young’s Modulus from hundreds of force curves of both copolymer synthesized in confinement and bulk samples are presented in Figure 3D–E. The strong influence of the nanostructures on this material can be seen. The nanopillars showed enhanced softening behavior (0.54 MPa) and, contrary to the nanostructured system, the stiffness of the non-nanostructured copolymer was considerably greater (9.9 MPa). 

**Figure 4:**
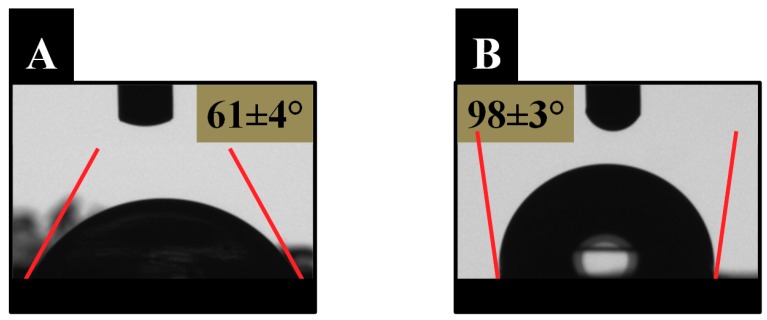
Water contact angle of P(BMA-HEA) free nanopillars of the copolymer synthesized in confinement (**A**) and films of the copolymer synthesized in bulk (**B**).

Finally, surface wettability also displayed significant changes if free nanopillars of copolymers synthesized in confinement are compared to films of copolymers synthesized in bulk. Figure 4 illustrates higher values of contact angle for the films of the copolymer synthesized in bulk (Figure 4B) with respect to free nanopillars of the copolymer synthesized in confinement (Figure 4A). These results are in agreement with the water affinity of these systems and their swelling capacity, and, as it has been previously demonstrated, an interpenetration effect in the nanostructured films is observed [10].

Our results show a good combination of mechanical characteristics, wettability and swelling properties. These features, along with the chemical characteristics of the monomers used, render the nanosystems obtained favorable for molecular design for applications in tissue engineering, drug delivery, and regenerative medicine, among others. In particular, material systems that incorporate softening polymers are very attractive due to the superior biocompatibility and high level of swellability. Their programmable large swelling capacity can be used in a diversity of environmentally responsive devices, microfluidic valves and artificial organs, and more.

## 4. Conclusion

A promising nanomaterial based on a butyl methacrylate-*co*-2-hydroxyethyl acrylate copolymer with confinement-induced softening was designed and prepared through free radical copolymerization in the nanocavities of anodized aluminum oxide templates.

The use of AAO nanoreactors to synthesize this copolymer under confinement produced interesting changes in the copolymer properties. To begin with, the copolymerization under confinement allowed to produce high polymer conversions with very controllable molecular weight and molecular weight dispersities, supporting our previous hypothesis, i.e., AAO template leads to a catalytic effect of AIBN and an increase in k_d_ value [8]. 

As far as the material properties are concerned, due to the nanostructure effect, the free nanopillars of the copolymer synthesized in confinement revealed a significant decrease in Young’s Modulus values as compared to copolymer films synthesized in bulk and a significant increase in their swelling capacity. 

This short communication is a valuable reference for obtaining a nanostructured copolymer in a single and fast step at a moderate temperature, thus, avoiding polymer degradation. In addition, the mechanical and wetting properties of these nanopillar structures could have valuable applications in surface science. Moreover, they could play a key role in the molecular design of polymer-based vehicles for tissue engineering, drug delivery, and regenerative medicine, among others. Indeed, the results of this work expand the applicability of AAO templates to pattern biological events, since natural polymers are developed within regulated and well-organized molecular nanoscale spaces, and, therefore, the AAO nanoreactor could be considered an important approach to synthesize polymers of biological interest.

## Supplementary Materials

The supplementary materials are available online at https://www.mdpi.com/2073-4360/11/2/290/s1. Figure S1: Top view from a huge crack of template polymerized “in-situ” with HEA-BMA, Figure S2: Extended H NMR spectra for homo- and copolymers obtained under bulk condition with assignments of resonance peaks.
