# Supplementary material for: A Patterned Butyl Methacrylate-co-2-Hydroxyethyl Acrylate Copolymer with Softening Surface and Swelling Capacity
Source: Polymers (Basel). 2019 Feb 9;11(2):290. doi: 10.3390/polym11020290 (PMC6419064; doi:10.3390/polym11020290)
Supplement: Supplementary file 1 [file polymers-11-00290-s001.pdf]

Supplementary Materials

A Patterned Butyl Methacrylate-*co*-2-Hydroxyethyl Acrylate Copolymer with Softening Surface and Swelling Capacity

Laia León-Boigues [1], Catalina von Bilderling [2,3], Lía I. Pietrasanta [3,4], Omar Azzaroni [2], Juan M. Giussi [2,*] and Carmen Mijangos [1,5,*]

[1] Instituto de Ciencia y Tecnología de Polímeros, ICTP-CSIC, Juan de la Cierva 3, 28006 Madrid, Spain; laleboi@ictp.csic.es
[2] Instituto de Investigaciones Fisicoquímicas Teóricas y Aplicadas (INIFTA)–Departamento de Química–Facultad de Ciencias Exactas-Universidad Nacional de La Plata–CONICET, 1900 La Plata–Argentina; catalinavb@inifta.unlp.edu.ar (C.v.B.); azzaroni@inifta.unlp.edu.ar (O.A.)
[3] Departamento de Física, Facultad de Ciencias Exactas y Naturales, Universidad de Buenos Aires, C1428EHA Buenos Aires, Argentina; lia@df.uba.ar
[4] Instituto de Física de Buenos Aires (IFIBA-CONICET), C1428EHA Buenos Aires, Argentina
[4] Donostia International Physics Center (DIPC), Paseo Manuel Lardizabal 4 and Centro de Fisica de Materiales, CFM-CSIC/UPV-EHU Paseo de Manuel Lardizabal 5, 20018 Donostia-San Sebastian, Spain
* Correspondence: jmgiussi@inifta.unlp.edu.ar (J.M.G.); cmijangos@ictp.csic.es (C.M.); Tel.: +54-0221-425-7291 (J.M.G.); +34-915-622-900 (C.M.)

Figure S1 shows a huge crack in AAO template with copolymer confined. Stretched filaments show the copolymer flexibility and stretching, and the polymer reaches to stretch up to 4 microns.

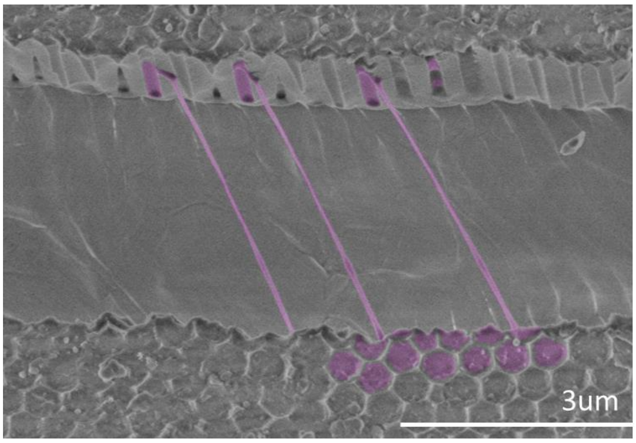

Figure S1. Top view from a huge crack of template polymerized "in-situ" with HEA-BMA.

Figure S2 shows the H NMR extended spectra between 3 and 5 ppm for the homo- and copolymers obtained under bulk conditions with the assignments of resonance peaks.

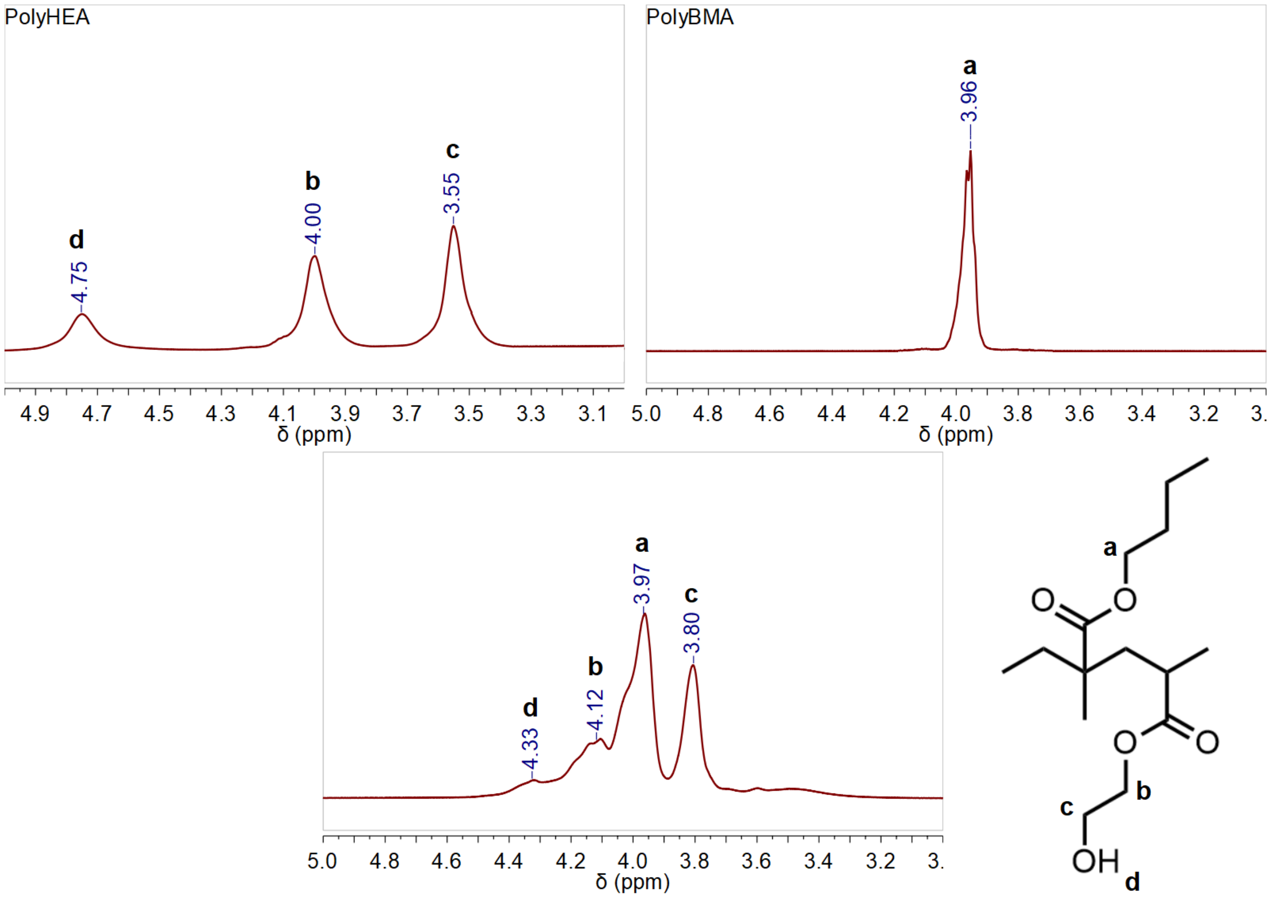

Figure S2. Extended H NMR spectra for homo- and copolymers obtained under bulk condition with assignments of resonance peaks.

The copolymer composition was estimated from the deconvolutionated H NMR signals (figure S2) at δ = 3.80 ppm (signal c for HEA monomer) and 3.97 ppm (signals a for BMA monomer), using Eq. S1.

$$F_{BMA} = \frac{I_{d=3.97}/2}{I_{d=3.97}/2 + I_{d=3.80}/2} \qquad \text{(eq S1)}$$

F_{BMA} represents the BMA molar fraction in the copolymers.

The final copolymer composition were 0.47 for BMA and 0.53 for HEA (F_{BMA}=0.47±0.3 and F_{HEA}=0.53±0.2) in copolymerization under confinement and 0.62 for BMA and 0.38 for HEA (F_{BMA}=0.60±0.5 and F_{HEA}=0.4±0.3) in copolymers obtained under bulk conditions.
